# Multi-gene panel testing for hereditary cancer predisposition in unsolved high-risk breast and ovarian cancer patients

**DOI:** 10.1007/s10549-017-4181-0

**Published:** 2017-03-09

**Authors:** Beth Crawford, Sophie B. Adams, Taylor Sittler, Jeroen van den Akker, Salina Chan, Ofri Leitner, Lauren Ryan, Elad Gil, Laura van ’t Veer

**Affiliations:** 1 University of California San Francisco, Helen Diller Family Comprehensive Cancer Center, 2340 Sutter Street, San Francisco, CA 94115 USA; 2 Stanford Children’s Health Center, Lucile Packard Children’s Hospital Stanford, 401 Quarry Rd, Stanford, CA 94305 USA; 3 Color Genomics, 128 Murchison Drive, Burlingame, CA 94010 USA; 4 Skypax LLC, 112 Timberlyne Court, Chapel Hill, NC USA

**Keywords:** Hereditary cancer, Panel testing, *BRCA1*, *BRCA2*, Breast cancer, Ovarian cancer

## Abstract

**Purpose:**

Many women with an elevated risk of hereditary breast and ovarian cancer have previously tested negative for pathogenic mutations in *BRCA1* and *BRCA2*. Among them, a subset has hereditary susceptibility to cancer and requires further testing. We sought to identify specific groups who remain at high risk and evaluate whether they should be offered multi-gene panel testing.

**Methods:**

We tested 300 women on a multi-gene panel who were previously enrolled in a long-term study at UCSF. As part of their long-term care, all previously tested negative for mutations in *BRCA1* and *BRCA2* either by limited or comprehensive sequencing. Additionally, they met one of the following criteria: (i) personal history of bilateral breast cancer, (ii) personal history of breast cancer and a first or second degree relative with ovarian cancer, and (iii) personal history of ovarian, fallopian tube, or peritoneal carcinoma.

**Results:**

Across the three groups, 26 women (9%) had a total of 28 pathogenic mutations associated with hereditary cancer susceptibility, and 23 women (8%) had mutations in genes other than *BRCA1* and *BRCA2*. Ashkenazi Jewish and Hispanic women had elevated pathogenic mutation rates. In addition, two women harbored pathogenic mutations in more than one hereditary predisposition gene.

**Conclusions:**

Among women at high risk of breast and ovarian cancer who have previously tested negative for pathogenic *BRCA1* and *BRCA2* mutations, we identified three groups of women who should be considered for subsequent multi-gene panel testing. The identification of women with multiple pathogenic mutations has important implications for family testing.

**Electronic supplementary material:**

The online version of this article (doi:10.1007/s10549-017-4181-0) contains supplementary material, which is available to authorized users.

## Introduction

The advent of next-generation sequencing (NGS) technology has revolutionized the clinical approach to genetic testing across many areas of medicine including medical oncology. Instead of single gene testing, interrogating a panel of multiple genes provides clinicians information about one or more disorders in a single test [1–4]. Additionally, new methods of identifying large rearrangements using NGS data have allowed for more comprehensive testing [5–7]. A number of studies have recently investigated the clinical validity of comprehensive multi-gene panels in the context of clinical management of breast and ovarian cancer [8–11]. These studies are clarifying which genes to include for each disease and how to counsel patients and their families regarding penetrance, screening, surveillance, and risk-reducing options.

Over one million people in the U.S. alone are believed to have had prior testing for pathogenic *BRCA1* and *BRCA2* mutations, but not a broader panel [12]. The great majority has received a negative test result, yet some still harbor an undiscovered pathogenic *BRCA1* or *BRCA2* mutation (due to limited sequencing) or a pathogenic mutation in another cancer susceptibility gene. Multiple studies have shown that 3–4% of high-risk individuals have germline pathogenic mutations in cancer risk genes other than *BRCA1* and *BRCA2*, including *ATM, CHEK2, PALB2, PTEN, TP53*, and others [4, 13]. With the emergence of broader multi-gene panels, re-testing these individuals will be required to identify those carrying previously unidentified mutations. However, no clear guidelines exist to suggest which individuals should be offered additional testing using such panels. In this study, we sought to identify characteristics among individuals previously negative for pathogenic *BRCA1* and *BRCA2* mutations who may benefit from multi-gene panel testing.

## Methods

### Study cohort

The UCSF-CGPP, previously named Cancer Risk Program, was founded in 1996 in order to provide genetic risk assessments for patients with personal and family histories of cancer. In 1997, the UCSF-CGPP received institutional review board approval for a long-term follow-up program aimed at promoting research efforts associated with hereditary cancer risk. The great majority of patients participating in the program banked one clinical sample for further family testing and one research sample. All patients who banked samples received genetic counseling and risk assessment. As of April, 2016, 7213 women had agreed to participate in this follow-up program and 4892 (68%) banked a DNA sample for research purposes. Of these, 1281 women (26%) had a personal history of breast or ovarian cancer, previously tested negative for pathogenic *BRCA1* or *BRCA2* mutations by one of several methods, and met NCCN criteria for *BRCA1* and *BRCA2* testing [14]. From this pool, we randomly selected de-identified and blinded samples which satisfied inclusion criteria listed in Table 1: bilateral breast cancer (*n* = 97), breast cancer and family history of ovarian cancer (*n* = 104), and ovarian cancer (*n* = 99). History of disease was confirmed by a breast oncologist via pathology review and/or medical record review (see supplementary eMethods). At the time of this study, 32 patients (10.7%) were confirmed deceased by family member notification or electronic medical record review.

**Table 1:** Study criteria

Inclusion criteria	Exclusion criteria
1. Female patient affected with	1. Previous clinical or research gene panel testing or whole exome sequencing
a. Bilateral breast cancer	
b. Breast cancer and a first-degree or second-degree relative with ovarian cancer	
c. Ovarian cancer (ovarian, fallopian tube cancer, or primary peritoneal carcinomatosis)	
2. Banked DNA sample for research	2. Previously identified pathogenic or likely pathogenic mutation in any gene
3. Met current (v1.2015) NCCN high-risk criteria	3. Adopted
4. Previously negative *BRCA1*/*BRCA2* testing (e.g., Ashkenazi Jewish founder mutations, *BRCA1/BRCA2* full sequencing, 5-site rearrangements, and/or deletion duplication)	4. <10 mcg DNA

*Legend* Inclusion and exclusion criteria used in this study

### Gene selection

The Color panel is comprised of 19 genes clinically relevant to breast and ovarian cancer: *ATM, BARD1, BRCA1, BRCA2, BRIP1, CDH1, CHEK2, EPCAM, MLH1, MSH2, MSH6, NBN, PALB2, PMS2, PTEN, RAD51C, RAD51D, STK11,* and *TP53.* Genes were selected based on published evidence that women who have pathogenic mutations in these genes are at increased risk of developing breast and/or ovarian cancer (see supplementary eTable 2). Preliminary studies have suggested that due to overlapping phenotypes, multi-gene panel testing will find a significant number of mutations that would be missed when testing for Hereditary Breast and Ovarian Cancer syndrome, Lynch syndrome, Cowden syndrome, Li-Fraumeni syndrome, or other hereditary cancer syndromes individually [15, 16]. For this reason, a single panel was created covering all of these syndromes rather than individual syndrome-specific panels.

### Gene sequencing

A total of 400 nanograms of banked genomic DNA were sheared on a Covaris LE-220 sonicator (Woburn, MA) to obtain 300 base pair (bp) mean size fragments. Genomic DNA was quantified and assessed for quality using DropSense UV spectroscopy as well as Biotium AccuBlue Fluorescence Assay (Ghent, Belgium; Hayward CA). The entire coding region, exon–intron boundaries (±20 bp), and other regions containing known pathogenic mutations were targeted and captured using Agilent SureSelect custom RNA probes (Santa Clara, CA). Sequencing libraries were constructed following the Agilent SureSelectXT protocol and were quantified using the KAPA Biosystems Library Quantification Kit (Woburn, MA). These steps were performed in an automated fashion using the Hamilton automated liquid-handling platform. Quantified libraries were sequenced on the Illumina NextSeq NGS platform (San Diego, CA) using the 2 × 150 bp configuration. Bioinformatics and data quality control followed the Genome Analysis Toolkit best practices (Broad Institute, Cambridge, MA), with additional algorithms to detect larger deletions and duplications. *PMS2* exons 12–15 were excluded from analysis because of high homology to a known pseudogene.

### Multi-gene panel validation

As part of this study, we validated the Color panel on 200 UCSF-CGPP patient samples harboring 159 *BRCA1* or *BRCA2* mutations and several mutations in additional genes associated with hereditary cancer. In this study, we validated the 200 samples using the Color multi-gene panel test prior to sequencing the 300 samples from our study cohort. The Color test correctly identified all previously observed clinically actionable mutations (supplementary eTable 3). In addition, four samples had two pathogenic mutations, the second of which was not identified by other laboratories in each case.

### Variant classification

Sequence variant classification as pathogenic, likely pathogenic, variant of uncertain significance (VUS), likely benign and benign was performed according to the American College of Medical Genetics and Genomics (ACMG) sequence variant interpretation guidelines [17]. All classifications were ultimately evaluated by a board-certified pathologist or medical geneticist. Likely benign and benign variants were not clinically reported. All variants classified as pathogenic, likely pathogenic, and VUS were confirmed via a secondary technique. Sanger sequencing was used to confirm single nucleotide variants (SNVs), and small insertions and deletions (indels), while larger deletions and duplications were confirmed via array comparative genomic hybridization (aCGH) or multiplex ligation-dependent probe amplification (MLPA).

## Results

### Patient demographics and cancer history

The study cohort consisted of 300 women who enrolled in UCSF-CGPP between 1999 and 2014 and satisfied the criteria described in Table 1. The majority of participants (*n* = 195, 65%) was Caucasian with a large proportion reporting Ashkenazi Jewish ancestry (*n* = 52, 17%). The rest of the cohort self-identified as Asian (*n* = 29, 10%), Hispanic (*n* = 22, 7%), unknown (*n* = 19, 6%), mixed racial background (*n* = 13, 4%), African (*n* = 10, 3%), Pacific Islander (*n* = 9, 3%), or Native American (*n* = 1, 0.3%) (Table 2).

**Table 2:** Demographics of the study population

	Personal history of bilateral breast cancer	Personal history of breast cancer, relative with ovarian cancer	Personal history of ovarian cancer	Cohort
Patients (n)	97	104	99	300
Mean age at first diagnosis	50 (28–72)	48 (23–77)	54 (19–80)	51 (19–80)
Race/ethnicity				
African	3%	4%	3%	3%
Ashkenazi	21%	16%	14%	17%
Asian	10%	8%	11%	10%
Caucasian	39%	55%	52%	49%
Hispanic	6%	8%	8%	7%
Mixed	4%	4%	5%	4%
Native American	0%	0%	1%	0.3%
Pacific Islands	2%	4%	3%	3%
Unknown	14%	2%	3%	6%

*Legend* Demographics reported by patients to genetic counselors as part of the UCSF-CGPP study

### Pathogenic mutations

Of the 300 high-risk women who had previously tested negative for *BRCA1* and *BRCA2* pathogenic mutations, 26 women had a total of 28 pathogenic mutations in at least one of the 19 genes sequenced, including three pathogenic *BRCA1* mutations that were not observed by previous, less comprehensive testing (Table 3a–c). The cumulative incidence of mutations in this cohort was 8.7% (*n* = 26) and was consistent (8–9%) across the three different high-risk groups. We observed two mutations in each of two women with a personal history of ovarian cancer (Table 3) and in four women in our validation set (Table 4).

**Table 3:** Mutations identified in our three study cohorts

Personal history	Gene	Pathogenic mutation(s)	Number of patients
Bilateral breast cancer	*ATM*	c.378delT	1
Bilateral breast cancer	*BRCA1*	deletion of exons 8–11 (deletion of exons 9–12)	1
Bilateral breast cancer	*CDH1*	c.1137G>A	1
Bilateral breast cancer	*CHEK2*	c.1100delC	5
Bilateral breast cancer	*CHEK2*	c.470T>C	1
Breast cancer, relative with ovarian cancer	*ATM*	c.742C>T	1
Breast cancer, relative with ovarian cancer	*BRCA1*	c.2125_2126insAGT (2244ins3)	1
Breast cancer, relative with ovarian cancer	*CHEK2*	c.1100delC	2
Breast cancer, relative with ovarian cancer	*PALB2*	c.172_175delTTGT	1
Breast cancer, relative with ovarian cancer	*PALB2*	c.2257C>T	1
Breast cancer, relative with ovarian cancer	*PALB2*	c.3323delA	1
Breast cancer, relative with ovarian cancer	*RAD51D*	c.270_271insAT, c.269_270dupAT	1
Ovarian cancer	*ATM*	c.2T>C	1
Ovarian cancer	*ATM*	c.5065C >T	1
Ovarian cancer	*ATM &* *PALB2*	c.901+1G >A c.2167_2168delAT	1
Ovarian cancer	*BRCA1*	c.5095C>T (R1699 W)	1
Ovarian cancer	*CHEK2*	c.1283C>T	1
Ovarian cancer	*CHEK2 &* *RAD51C*	c.1100delC c.397C>T	1
Ovarian cancer	*MSH6*	c.3438+1G>C	1
Ovarian cancer	*NBN*	c.1397+1delG	1
Ovarian cancer	*PALB2*	c.2457delA	1

*Legend* Pathogenic variants found using the Color panel in our study cohort

**Table 4:** Validation samples with more than one pathogenic mutation

Gene	Pathogenic mutations	Number of patients
*BARD1 & BRCA1*	c.1996C>T (Q666*) c.1687C>T (Q563X)	1
*BRCA1 & BRCA1*	deletion of exons 7–9, and part of exon 10 (deletion of exons 8–10, and part of exon 11) c.2101A>T (K701X)	1
*BRCA2 & CHEK2*	deletion of exon 3 c.499G>A	1
*PALB2 & PMS2*	c.172_175delTTGT c.400C>T (R134*)	1
Total patients		4

*Legend* Summarizes women in the validation set with more than one pathogenic mutation identified (complete list in supplementary eTable 3)

### Personal history of bilateral breast cancer

A total of 99 women in the study cohort had a personal history of bilateral breast cancer or multiple breast cancers with at least one in the contralateral breast. The average age at diagnosis of the first breast cancer was 50, consistent with the average age for the overall cohort. We detected pathogenic mutations in nine of the 99 women in this group involving the *ATM, BRCA1, CDH1*, and *CHEK2* genes, all of which have been associated with an increased risk of bilateral breast cancer in the previous studies [18–22]. There were a disproportionately large number of *CHEK2* c.1100delC mutations which have been postulated to be enriched in bilateral breast cancer cases [22, 23]. Of note, one large *BRCA1* deletion common in the Hispanic population was observed in a woman who previously received negative test results for pathogenic *BRCA1* and *BRCA2* mutations (Table 3). Investigation of her previous testing revealed that large deletions and duplications had not been assayed.

### Personal history of breast cancer and family history of ovarian cancer

A total of 104 women in the study cohort had a personal history of breast cancer and a family history of ovarian cancer. The average age at diagnosis was 48. Eight of 104 women had pathogenic mutations, one of which was a previously undetected pathogenic *BRCA1* mutation in a Hispanic woman who had limited mutation testing (Table 3). Most pathogenic mutations were in genes with an established breast cancer risk (*ATM, BRCA1, CHEK2, and PALB2*). Interestingly, an additional mutation was detected in *RAD51D*, which may be associated with increased risk of breast cancer and is associated with increased risk of ovarian cancer [11, 24, 25].

### Personal history of ovarian, fallopian tube cancer, or peritoneal carcinomatosis

A total of 97 women in the study cohort had a personal history of ovarian, fallopian tube cancer, or peritoneal carcinomatosis. The average age at diagnosis of cancer was 54. Nine of 97 women had pathogenic mutations. Two women in this group had two pathogenic mutations each, in two different genes: one had pathogenic mutations in *CHEK2* and *RAD51C,* and the other had pathogenic mutations in *ATM* and *PALB2*. Pathogenic mutations were observed in several genes with well-established ovarian cancer risk: *MSH6*, *NBN*, *RAD51C*, and one mutation in *BRCA1* that was previously undetected due to limited testing. We also observed pathogenic mutations in several genes that have not traditionally been associated with increased risk of ovarian cancer (*PALB2*, *ATM*, and *CHEK2*).

## Discussion

### Considerations for panel testing

Today, genetic testing for Hereditary Breast Ovarian Cancer syndrome has moved from testing of the *BRCA1* and *BRCA2* genes to broader panel testing. Here we focused on clinical considerations for panel testing in women who had previously tested negative for *BRCA1* and *BRCA2* mutations. We studied three such groups: (i) women with a personal history of bilateral breast cancer, (ii) women with a personal history of breast cancer and a first-degree or second-degree relative with ovarian cancer, and (iii) women with a personal history of ovarian, fallopian tube, or peritoneal carcinoma. From our study of these groups, three criteria for re-testing emerged. First, history of breast and ovarian cancer consistent with any of the groups enumerated above suggests an elevated risk of hereditary cancer and multi-gene panel testing for additional susceptibility genes should be considered. Second, individuals who have previously received limited *BRCA1* and *BRCA2* gene testing may still harbor a genetic risk of breast and/or ovarian cancer and should be considered for multi-gene panel testing including large rearrangement testing. Third, the presence of individuals with multiple pathogenic mutations in both cohort samples and validation samples, consistent with the previous studies [9], suggests that comprehensive multi-gene panel testing could supplant targeted testing for single known familial mutations.

### History of cancer

Personal history of breast cancer, with and without a family history of ovarian cancer, was the primary criteria used to select individuals for this study from among all those who had previously tested negative for *BRCA1* and *BRCA2* mutations. We observed an 8–9% pathogenic mutation rate (Table 3) including previously missed *BRCA1* mutations, and a 7–8% pathogenic rate not including *BRCA1* mutations. This rate is two to three times the rate of pathogenic mutations previously reported in these genes among women with breast cancer or among individuals with significant family history of breast cancer alone (3–4%) [4, 13], indicating that this group is enriched for pathogenic mutations associated with hereditary breast and ovarian cancer, hereditary diffuse gastric cancer, or Lynch syndrome genes compared to high-risk individuals in general. The high pathogenic mutation rate in this cohort suggests that all individuals who meet criteria for inclusion in one of our subgroups would benefit from multi-gene panel testing.

In addition to these groups, there likely exist broader groups of the previously tested individuals who harbor pathogenic mutations that warrant re-testing using multi-gene panels. For instance, the high rate of mutations we observed in women who met NCCN criteria and also had personal history of ovarian cancer (8%) could indicate that all women with ovarian cancer may benefit from multi-gene panel testing. Supporting this hypothesis, previous studies have demonstrated elevated rates of pathogenic mutations in genes other than *BRCA1* and *BRCA2* (6–7%) [3, 8] among women with ovarian cancer. The NCCN and the Society of Gynecologic Oncology both recommend *BRCA1* and *BRCA2* genetic testing for all women with ovarian cancer, and parallel re-testing of all of these women using multi-gene panels may be warranted due to the observed mutation rates in this group.

### Limited genetic testing and ethnicity

In the study cohort, three types of limited *BRCA1* and *BRCA2* testing were previously used by other laboratories: Ashkenazi founder mutation testing, Hispanic founder mutation testing, and gene testing without analysis of large deletions and duplications. Multi-gene panel testing by Color panel identified three mutations in the *BRCA1* gene (12% of mutations in the study cohort) that were not identified by the previous limited testing. One pathogenic *BRCA1* mutation was identified in a woman who had testing limited to Ashkenazi Jewish and Hispanic mutations [26] due to her combined ancestry; one mutation was identified in a woman who had testing of only Hispanic mutations; and one mutation was identified in a woman who had previous full sequencing of *BRCA1* and *BRCA2* without assessment of large deletions and duplications.

All three of these mutations were missed in individuals of partial or full Hispanic origin. Given the increase in frequency of large deletions and duplications in the Hispanic population [27–29] (20% of identified mutations in high-risk Hispanic populations) and the relatively recent lack of testing available for such variants [26, 30, 31], missed mutations in these genes may be more common in the high-risk Hispanic population than in other ethnicities.

Additionally, elevated rates of pathogenic mutations in other genes were observed in certain ethnicities, particularly among Ashkenazi Jewish and Hispanic individuals (12 and 18%, respectively, see Table 5). It appears that limited *BRCA1* and *BRCA2* testing based on ethnicity may miss a significant number of clinically actionable mutations. There may be a larger range of mutations expressed in a single ethnicity than previously observed [4, 32], and this effect may be exacerbated by reported or unreported mixed ethnic backgrounds. Because of the potential to miss such mutations, re-testing of individuals who previously had limited *BRCA1* and *BRCA2* testing should be considered, particularly in ethnicities with elevated risk or if a strong suspicion for hereditary cancer otherwise remains. Further, with individuals of unclear ancestry, unknown ancestry, or mixed-race, multi-gene panel testing could be considered as the primary test for identification of mutations associated with hereditary cancer susceptibility.

**Table 5:** Distribution of mutations within each ethnicity

	Personal history of bilateral breast cancer (%)	Personal history of breast cancer, relative with ovarian cancer (%)	Personal history of ovarian cancer (%)	Cohort (%)
Race/ethnicity				
African	0	25	0	10
Ashkenazi	10	6	21	12
Asian	0	13	9	7
Caucasian	16	5	4	8
Hispanic	17	13	25	18
Pacific Islands	0	25	20	15
Group cumulative	9	9	8	8.7

*Legend* Pathogenic variants identified in each reported ethnicity within each group and in the whole cohort

### Family testing of mutation carriers

Following identification of a pathogenic mutation, a carrier’s family members are typically offered targeted single-site testing (or cascade testing) specific to the identified pathogenic mutation in order to determine each member’s risk of hereditary cancer. The identification of pathogenic mutations in two different genes in a single individual, present in both our validation set and study cohort, indicates that such testing may be inadequate to clinically determine the risk of hereditary cancer for two reasons:

1. If a mutation carrier has two mutations and those mutations are expected to segregate separately, family members of the carrier who test negative by single-site testing for one of the mutations may still carry the other.
2. If a carrier is discovered with a mutation in a single gene, family members may carry a different mutation (in a different gene) inherited separately, whether that member tests positive or negative for the previously discovered family mutation.

Pathogenic mutations in multiple genes in the same individual have been observed in approximately 3% of patients in larger cohorts who received breast, ovarian, colon, and general hereditary cancer risk testing [9], similar to the 1–2% of multiple mutation carriers identified within our study. These findings indicate that individuals with multiple mutations are identified with some frequency and their family members may be falsely reassured based on single-site testing alone. Because of this issue, multi-gene panel testing should be considered to identify these missed pathogenic mutations, providing a more accurate assessment of hereditary cancer risk in known mutation carriers and their family members.

### Testing options

Despite negative *BRCA1* and *BRCA2* test results, in certain cases, clinicians often remain suspicious of another hereditary cancer syndrome due to the family history of cancer. Nevertheless, efforts to obtain additional genetic testing are often limited due to lack of insurance coverage, resulting in prohibitively high costs for patients. With the rise of more affordable testing options, clinicians and their patients now have greater access to multi-gene panel testing, both as a follow-on test for those with previously incomplete testing and as a first-line approach.

### Limitations and future directions

This study was enriched for individuals at high risk of breast and ovarian cancer as defined by the NCCN criteria. Consequently, this study cohort likely represents individuals with higher than average breast and ovarian cancer risks and is not representative of those patients with mild to moderate cancer risks nor is it representative of the general population. Larger cohorts will be required to determine more accurate rates of pathogenic mutations in women with previously negative hereditary cancer testing.

The study population represented a cohort of primarily Caucasian women, which is not generalizable to the population at large. Furthermore, a large proportion (17%) of patients in the cohort were of Ashkenazi Jewish descent. While other studies have found no increased detection rate despite enrichment for Ashkenazi Jewish participants [4, 13], the high proportion of Ashkenazi Jewish women in our study may have altered the number or type of pathogenic variants detected.

## Electronic supplementary material

Below is the link to the electronic supplementary material.

Supplementary material 1 (DOCX 33 kb)
