# Supplementary material for: Multi-gene panel testing for hereditary cancer predisposition in unsolved high-risk breast and ovarian cancer patients
Source: Breast Cancer Res Treat. 2017 Mar 9;163(2):383–90. doi: 10.1007/s10549-017-4181-0 (PMC5410210; doi:10.1007/s10549-017-4181-0)
Supplement: Supplementary file 1 — Supplementary material 1 (DOCX 33 kb) [file 10549_2017_4181_MOESM1_ESM.docx]

SUPPLEMENT

MULTI-GENE PANEL TESTING FOR HEREDITARY CANCER PREDISPOSITION IN UNSOLVED HIGH RISK BREAST AND OVARIAN CANCER PATIENTS

# Authors

Beth Crawford, MS^a^, Sophie B. Adams, MS^a,b^, Taylor Sittler, MD^a,c^, Jeroen van den Akker, PhD^c^, Salina Chan, BS^a^, Ofri Leitner, MS^d^, Lauren Ryan, MS^a,c^, Elad Gil, PhD^c^, Laura van't Veer, PhD^a^

^a^ University of California San Francisco

^b^ Stanford Children’s Health Center | Lucile Packard Children's Hospital Stanford

^c^ Color Genomics, Burlingame, CA

^d^ Skypax, LLC, Chapel Hill, NC

#

#

#

# eMethods

## Data Collection

Clinical data was gleaned from electronic medical record review, paper charts, and a 16-page questionnaire that queries physiological, environmental, and genetic risk factors for cancer. In addition, a four-generation family history was collected by a certified genetic counselor at the time of the patient’s appointment as part of the University of California San Francisco Cancer Genetics and Prevention Program (UCSF-CGPP) protocol. When possible, genetic counselors collected medical records, death certificates, and genetic testing reports to corroborate family history information. Pedigree data was entered using GenoSketch and Progeny Software in order to maintain family structure and track the number of unaffected family members. This data was not shared with Color Genomics prior to sequencing results were received by UCSF.

## Family History Information

At the time of the patient’s appointment, a genetic counselor or medical geneticist elicited a four-generation pedigree. Typically, the medical history of fourth-degree relatives is not documented unless an individual is affected.

# eTable 1: Cancers By Group

|  |  |  |  |  |
| --- | --- | --- | --- | --- |
|  | **PERSONAL HISTORY OF BILATERAL BREAST CANCER** | **PERSONAL HISTORY OF BREAST CANCER, RELATIVE WITH OVARIAN CANCER** | **PERSONAL HISTORY OF OVARIAN CANCER** | **COHORT** |
| Bilateral breast | 97 |  | 1 | 95 |
| Breast |  | 104 | 25 | 217 |
| Fallopian tube |  |  | 9 | 9 |
| Ovary |  |  | 88 | 96 |
| Peritoneum |  |  | 3 | 3 |
| **Study Cancer Total** | **97** | **104** | **99** | **300** |
| Adipose |  |  | 1 | 1 |
| Adrenal | 1 |  |  | 1 |
| Bladder |  | 1 | 1 | 2 |
| Bone | 1 | 2 | 1 | 4 |
| Brain | 2 | 4 |  | 6 |
| Cervix |  | 1 | 1 | 2 |
| Chest wall | 1 |  |  | 1 |
| Colorectal | 3 | 3 | 3 | 9 |
| Leukemia |  | 1 |  | 1 |
| Liver |  | 2 | 1 | 3 |
| Lung | 1 | 2 |  | 3 |
| Lymph Node | 3 |  | 1 | 4 |
| Pancreas | 1 |  |  | 1 |
| Parathyroid |  | 2 |  | 2 |
| Parotid | 1 | 1 |  | 2 |
| Pituitary | 1 | 1 |  | 2 |
| Skin | 6 | 2 | 9 | 17 |
| Spine |  | 1 |  | 1 |
| Thyroid |  | 1 |  | 1 |
| Uterus | 2 |  | 5 | 7 |
| Vulva |  |  | 1 | 1 |
| Other Cancer Total | **19** | **19** | **22** | 60 |

## Cancer Pathology

Our cohort consisted of women with three different types of cancer, summarized in the patient accrual section. for bilateral breast cancer 50, and for those with a family history of ovarian cancer and unilateral breast cancer it was 48, with an overall average of 50 years for the cohort. Among patients with breast cancer (unilateral and bilateral), 49 had invasive pathology of unclear type and 17 had in situ disease of unclear type. These cancers were labeled “carcinoma” or “carcinoma in situ”. If there were features of both ductal and lobular carcinoma, these were also labeled “carcinoma”. If both unilateral and bilateral breast cancer were diagnosed at the same age, the cancer was presumed to be bilateral and the phenotype was combined.

Of the 99 women in group A, nine had fallopian tube cancer and three had primary peritoneal cancer. Twenty-six patients in group A also had breast cancer, one of which had bilateral breast cancer. Most other cancers were equally distributed among the groups with the exception of skin, uteran, and brain cancers.

#

#

# eTable 2: Gene-disease associations

| **GENE** | **INHERITANCE** | **CANCER SYNDROME / SUSCEPTIBILITY** | **REFERENCES** |
| --- | --- | --- | --- |
| ***ATM*** | Recessive  Dominant | Ataxia-Telangiectasia  Breast cancer susceptibility | [(Easton et al. 2015)](https://paperpile.com/c/SqcMU7/ZHge)  [(Roberts et al. 2012)](https://paperpile.com/c/SqcMU7/JCgE) |
| ***BARD1*** | Dominant | Breast cancer susceptibility | [(De Brakeleer et al. 2010)](https://paperpile.com/c/SqcMU7/69iV)  [(Couch et al. 2015)](https://paperpile.com/c/SqcMU7/v3vY) |
| ***BRCA1*** | Dominant | Hereditary Breast and Ovarian Cancer syndrome | [(King et al. 2003)](https://paperpile.com/c/SqcMU7/KEbA)  [(Tai et al. 2007)](https://paperpile.com/c/SqcMU7/wNcs)  [(Liede et al. 2004)](https://paperpile.com/c/SqcMU7/9Vlk)  [(Mocci et al. 2013)](https://paperpile.com/c/SqcMU7/Takl)  [(Leongamornlert et al. 2012)](https://paperpile.com/c/SqcMU7/maUg) |
| ***BRCA2*** | Recessive  Dominant | Fanconi anemia  Hereditary Breast and Ovarian Cancer syndrome  Melanoma susceptibility | [(King et al. 2003)](https://paperpile.com/c/SqcMU7/KEbA)  [(Tai et al. 2007)](https://paperpile.com/c/SqcMU7/wNcs)  [(Liede et al. 2004)](https://paperpile.com/c/SqcMU7/9Vlk)  [(Mocci et al. 2013)](https://paperpile.com/c/SqcMU7/Takl)  [(Leongamornlert et al. 2012)](https://paperpile.com/c/SqcMU7/maUg)  [(Consortium and Others 1999)](https://paperpile.com/c/SqcMU7/Mhlp)  [(van Asperen et al. 2005)](https://paperpile.com/c/SqcMU7/LTzo) |
| ***BRIP1*** | Recessive  Dominant | Fanconi anemia  Breast cancer susceptibility | [(Seal et al. 2006)](https://paperpile.com/c/SqcMU7/2URF)  [(Easton et al. 2015)](https://paperpile.com/c/SqcMU7/ZHge)  [(Walsh et al. 2011)](https://paperpile.com/c/SqcMU7/E6mZ)  [(Rafnar et al. 2011)](https://paperpile.com/c/SqcMU7/ybcJ) |
| ***CDH1*** | Dominant | Hereditary diffuse gastric cancer | [(Hansford et al. 2015)](https://paperpile.com/c/SqcMU7/yrWU)  [(Pharoah et al. 2001)](https://paperpile.com/c/SqcMU7/joLA) |
| ***CHEK2*** | Dominant | Breast cancer susceptibility  Colorectal cancer susceptibility | [(Cybulski et al. 2011)](https://paperpile.com/c/SqcMU7/PqBu)  [(Weischer et al. 2008)](https://paperpile.com/c/SqcMU7/hDN7)  [(Xiang et al. 2011)](https://paperpile.com/c/SqcMU7/bGVp)  [(Cybulski et al. 2006)](https://paperpile.com/c/SqcMU7/sbIO)  [(Baysal et al. 2004)](https://paperpile.com/c/SqcMU7/B8Xz) |
| ***EPCAM*** | Dominant | Lynch syndrome | [(Bonadona et al. 2011)](https://paperpile.com/c/SqcMU7/uBcE)  [(Dowty et al. 2013)](https://paperpile.com/c/SqcMU7/QR27)  [(Barrow et al. 2009)](https://paperpile.com/c/SqcMU7/Zgsf)  [(Watson et al. 2008)](https://paperpile.com/c/SqcMU7/dHeN)  [(South et al. 2008)](https://paperpile.com/c/SqcMU7/AU5f)  [(Kastrinos et al. 2009)](https://paperpile.com/c/SqcMU7/JHh6) |
| ***MLH1*** | Recessive  Dominant | Constitutional mismatch repair deficiency syndrome (CMMR-D)  Lynch syndrome | [(Bonadona et al. 2011)](https://paperpile.com/c/SqcMU7/uBcE)  [(Dowty et al. 2013)](https://paperpile.com/c/SqcMU7/QR27)  [(Barrow et al. 2009)](https://paperpile.com/c/SqcMU7/Zgsf)  [(Watson et al. 2008)](https://paperpile.com/c/SqcMU7/dHeN)  [(South et al. 2008)](https://paperpile.com/c/SqcMU7/AU5f)  [(Kastrinos et al. 2009)](https://paperpile.com/c/SqcMU7/JHh6) |
| ***MSH2*** | Recessive  Dominant | CMMR-D  Lynch syndrome | [(Bonadona et al. 2011)](https://paperpile.com/c/SqcMU7/uBcE)  [(Dowty et al. 2013)](https://paperpile.com/c/SqcMU7/QR27)  [(Barrow et al. 2009)](https://paperpile.com/c/SqcMU7/Zgsf)  [(Watson et al. 2008)](https://paperpile.com/c/SqcMU7/dHeN)  [(South et al. 2008)](https://paperpile.com/c/SqcMU7/AU5f)  [(Kastrinos et al. 2009)](https://paperpile.com/c/SqcMU7/JHh6) |
| ***MSH6*** | Recessive  Dominant | CMMR-D  Lynch syndrome | [(Bonadona et al. 2011)](https://paperpile.com/c/SqcMU7/uBcE)  [(Barrow et al. 2009)](https://paperpile.com/c/SqcMU7/Zgsf)  [(Baglietto et al. 2010)](https://paperpile.com/c/SqcMU7/XygJ)  [(Kastrinos et al. 2009)](https://paperpile.com/c/SqcMU7/JHh6) |
| ***NBN*** | Recessive  Dominant | Breast cancer susceptibility  Prostate cancer susceptibility | [(Zhang et al. 2013)](https://paperpile.com/c/SqcMU7/CNYC)  [(Zhang et al. 2011)](https://paperpile.com/c/SqcMU7/lQtN)  [(Cybulski et al. 2004)](https://paperpile.com/c/SqcMU7/GWAE) |
| ***PALB2*** | Recessive  Dominant | Fanconi Anemia  Breast cancer susceptibility  Ovarian cancer  Pancreatic cancer susceptibility | [(Antoniou et al. 2014)](https://paperpile.com/c/SqcMU7/lyZ5)  [(Casadei et al. 2011)](https://paperpile.com/c/SqcMU7/bbjd)  [(Walsh et al. 2011)](https://paperpile.com/c/SqcMU7/E6mZ)  [(Becker et al. 2014)](https://paperpile.com/c/SqcMU7/rUyx) |
| ***PMS2*** | Recessive  Dominant | CMMR-D  Lynch syndrome | [(ten Broeke et al. 2015)](https://paperpile.com/c/SqcMU7/AjQd)  [(Senter et al. 2008)](https://paperpile.com/c/SqcMU7/I3uX)  [(Win et al. 2013)](https://paperpile.com/c/SqcMU7/5Iqa) |
| ***PTEN*** | Dominant | Cowden syndrome | [(Tan et al. 2012)](https://paperpile.com/c/SqcMU7/AMdz)  [(Bubien et al. 2013)](https://paperpile.com/c/SqcMU7/J12V) |
| ***RAD51C*** | Recessive  Dominant | Fanconi anemia  Ovarian cancer susceptibility | [(Loveday et al. 2012)](https://paperpile.com/c/SqcMU7/QoNs) |
| ***RAD51D*** | Dominant | Ovarian cancer susceptibility | [(Loveday et al. 2011)](https://paperpile.com/c/SqcMU7/jCrc) |
| ***STK11*** | Dominant | Peutz-Jegher syndrome | [(Lim et al. 2004)](https://paperpile.com/c/SqcMU7/uVuK)  [(Hearle et al. 2006)](https://paperpile.com/c/SqcMU7/gEUH)  [(Giardiello et al. 2000)](https://paperpile.com/c/SqcMU7/Iu7U)  [(van Lier et al. 2010)](https://paperpile.com/c/SqcMU7/1or6) |
| ***TP53*** | Dominant | Li-Fraumeni syndrome | [(Hwang et al. 2003)](https://paperpile.com/c/SqcMU7/vtfu)  [(Ruijs et al. 2010)](https://paperpile.com/c/SqcMU7/CEQj)  [(Olivier et al. 2003)](https://paperpile.com/c/SqcMU7/QDHZ)  [(Masciari et al. 2011)](https://paperpile.com/c/SqcMU7/K9BF)  [(Wong et al. 2006)](https://paperpile.com/c/SqcMU7/DZUa) |

Published evidence of breast and ovarian cancer disease associations in the 19 genes tested.

###

#

#

# eTable 3: Validation of Gene Panel

| **Gene** | **Mutation** | **Number of Patients** | **Color result matches previous observations** |
| --- | --- | --- | --- |
| ATM | c.2251-10T>G | 1 | Yes |
|  | c.3931C>T | 1 | Yes |
|  | c.4303A>T | 1 | Yes |
|  | c.9022C>T | 1 | Yes |
| BARD1 &  BRCA1 | c.1996C>T*  c.1687C>T (Q563X) | 1 | Partial* |
| BRCA1 | c.1016dupA (1135insA, 1127insA) | 1 | Yes |
|  | c.1175_1214del40 (1294del40) | 1 | Yes |
|  | c.1390_1391insG | 1 | Yes |
|  | c.1504_1508delTTAAA (1623del5) | 1 | Yes |
|  | c.181T>G (300T>G, C61G) | 1 | Yes |
|  | c.1921_1922insA (2040insA) | 2 | Yes |
|  | c.1961_1962insA (2071insA) | 1 | Yes |
|  | c.2071delA (2187delA) | 2 | Yes |
|  | c.2105dupT (2224insT) | 1 | Yes |
|  | c.211T>C (R71G) | 3 | Yes |
|  | c.2125_2126insAGT (2244ins3) | 1 | Yes |
|  | c.2269delG (2388delG) | 1 | Yes |
|  | c.2309C>A (S770X) | 1 | Yes |
|  | c.2457delC (2576delC) | 1 | Yes |
|  | c.2681_2682delAA (2800delAA) | 2 | Yes |
|  | c.2806_2809delGATA (2925del4) | 2 | Yes |
|  | c.2866_2870delTCTCA (2982del5) | 1 | Yes |
|  | c.2999delA (3118delA) | 1 | Yes |
|  | c.3008_3009delTT (3127delTT) | 1 | Yes |
|  | c.302-2A>C (IVS6-2A>C) | 1 | Yes |
|  | c.3029_3030delCT (3148delCT) | 1 | Yes |
|  | c.3358_3359delGT (3477delGT) | 1 | Yes |
|  | c.3627dupA (3627dupA, 3746dupA, 3746insA) | 1 | Yes |
|  | c.3700_3704delGTAAA (3819del5) | 1 | Yes |
|  | c.3756_3759delGTCT (3874del4) | 3 | Yes |
|  | c.3759dupT | 1 | Yes |
|  | c.4065_4068delTCAA (4184del4) | 1 | Yes |
|  | c.4096+1G>A (IVS11+1G>A) | 1 | Yes |
|  | c.427G>T (E143X) | 1 | Yes |
|  | c.4484G>T (R1495M) | 3 | Yes |
|  | c.4986+1G>T (IVS16+1G>T) | 1 | Yes |
|  | c.5074C>T (D1692N) | 1 | Yes |
|  | c.5095C>T (R1699W) | 1 | Yes |
|  | c.5153-2delA (IVS18-2delA) | 1 | Yes |
|  | c.5207T>C (V1736A) | 1 | Yes |
|  | c.5266dupC (5385insC, 5382insC) | 1 | Yes |
|  | c.5277+1G>A (IVS20+1G>A) | 1 | Yes |
|  | c.5332+1delG (IVS21+1delG) | 1 | Yes |
|  | c.5335delC (5454delC) | 1 | Yes |
|  | c.5363G>T (G1788V) | 1 | Yes |
|  | c.5503C>T (R1835X) | 1 | Yes |
|  | c.68_69delAG (187delAG, 185delAG) | 5 | Yes |
|  | c.798_799delTT (916delTT) | 2 | Yes |
|  | c.815_824dupAGCCATGTGG (943ins10, 934ins10, 934_943dup10) | 1 | Yes |
|  | deletion of exon 12 (deletion of exon 13) | 1 | Yes |
|  | deletion of exons 1-2 | 1 | Yes |
|  | deletion of exons 12-14 (deletion of exons 13 to 15) | 1 | Yes |
|  | deletion of exons 7-12 (deletion of exons 8 to 13) | 1 | Yes |
|  | deletion of exons 8-11 (deletion of exons 9-12) | 2 | Yes |
| BRCA1 &  BRCA1 | deletion of exons 7-9, and part of exon 10 (deletion of exons 8-10, and part of exon 11)  c.2101A>T (K701X)* | 1 | Partial* |
| BRCA2 | c.1205delG (1433delG) | 1 | Yes |
|  | c.1238delT (1466delT) | 1 | Yes |
|  | c.145G>T (E49X) | 1 | Yes |
|  | c.1581delA (1809delA) | 1 | Yes |
|  | c.1800T>A (Y600X) | 1 | Yes |
|  | c.1814dupA (2041insA, 2040insA, 2034insA) | 2 | Yes |
|  | c.1832C>A (S611X) | 1 | Yes |
|  | c.1850C>G (S617X) | 1 | Yes |
|  | c.1929delG (2157delG) | 1 | Yes |
|  | c.2098delT | 1 | Yes |
|  | c.2150delG (2378delG) | 1 | Yes |
|  | c.2466delT (2694delT) | 1 | Yes |
|  | c.2808_2811delACAA (3036del4, 3034del4) | 3 | Yes |
|  | c.2979G>A (W993X) | 2 | Yes |
|  | c.3160_3163delGATA (3388del4) | 1 | Yes |
|  | c.3264dupT (3492insT) | 5 | Yes |
|  | c.3358delG (3586delG) | 1 | Yes |
|  | c.3847_3848delGT (4075delGT) | 2 | Yes |
|  | c.3975_3976insTGCT (4206ins4) | 1 | Yes |
|  | c.3delG (231delG) | 1 | Yes |
|  | c.4012delG (4241delG) | 1 | Yes |
|  | c.4276dupA (4504insA) | 1 | Yes |
|  | c.4404_4408delTGACA (4633del5) | 1 | Yes |
|  | c.4631delA (4859delA) | 2 | Yes |
|  | c.4631dupA (4859insA) | 1 | Yes |
|  | c.470dupA (698insA, 698dup) | 1 | Yes |
|  | c.4889C>G (S1630X) | 1 | Yes |
|  | c.4936_4939delGAAA (5164del4) | 1 | Yes |
|  | c.4965C>G (Y1655X) | 1 | Yes |
|  | c.5073dupA (5301insA) | 1 | Yes |
|  | c.5164_5165delAG (5392delAG) | 1 | Yes |
|  | c.517-2A>G (IVS6-2A>G) | 1 | Yes |
|  | c.5213_5216delCTTA (5441del4) | 1 | Yes |
|  | c.5238dupT (5466insT) | 1 | Yes |
|  | c.5351dupA (5579insA) | 1 | Yes |
|  | c.5471dupA (5699dupA) | 1 | Yes |
|  | c.5609_5610delTCinsAG (F1870X) | 1 | Yes |
|  | c.5782G>T (E1928X) | 1 | Yes |
|  | c.5946delT (6174delT) | 8 | Yes |
|  | c.5980C>T (Q1994X) | 2 | Yes |
|  | c.6059_6062delAACA (6287del4) | 2 | Yes |
|  | c.6082_6086delGAAGA (6310del5) | 1 | Yes |
|  | c.6275_6276delTT (6503delTT) | 1 | Yes |
|  | c.631+2T>G (IVS7+2T>G) | 2 | Yes |
|  | c.631G>C (V211L) | 2 | Yes |
|  | c.6638delC (6866delC) | 1 | Yes |
|  | c.700delT (928delT, 924delT) | 1 | Yes |
|  | c.7069_7070delCT (7297delCT) | 2 | Yes |
|  | c.7133C>G (S2378X) | 1 | Yes |
|  | c.7377_7380delAAAC (7607del4) | 3 | Yes |
|  | c.7558C>T (R2520X) | 1 | Yes |
|  | c.7977-1G>C (IVS17-1G>C) | 1 | Yes |
|  | c.8009C>T (S2670L) | 1 | Yes |
|  | c.8253dupT (8481insT) | 1 | Yes |
|  | c.8297delC (8525delC) | 3 | Yes |
|  | c.8537_8538delAG (8765delAG) | 1 | Yes |
|  | c.8585dupT (8813insT) | 1 | Yes |
|  | c.8903delC (9132delC) | 1 | Yes |
|  | c.9076C>T (Q3026X) | 1 | Yes |
|  | c.9235delG (9463delG) | 1 | Yes |
|  | c.9294C>A (Y3098X) | 1 | Yes |
|  | c.9380G>A (W3127X) | 1 | Yes |
|  | c.9501+3A>T (IVS25+3A>T) | 1 | Yes |
| BRCA2 &  CHEK2 | deletion of exon 3  c.499G>A* | 1 | Partial* |
| CDH1 | c.1565+1G>T | 1 | Yes |
| CHEK2 | c.1100delC | 2 | Yes |
|  | c.1555C>T | 1 | Yes |
| MLH1 | c.1852_1854delAAG | 1 | Yes |
|  | c.2011G>T | 1 | Yes |
|  | deletion of exon 14 | 1 | Yes |
| PALB2 | c.599delT | 1 | Yes |
| PALB2 &  PMS2 | c.172_175delTTGT  c.400C>T* | 1 | Partial* |
| RAD51C | c.236dupT | 1 | Yes |
| RAD51D | c.345+2T>C | 1 | Yes |
|  | c.81delA | 1 | Yes |
| TP53 | c.1010G>T | 1 | Yes |
|  | c.1101-2A>G | 1 | Yes |
|  | c.524G>A | 1 | Yes |
|  | c.743G>A | 1 | Yes |
|  | c.799C>T | 1 | Yes |

* Second mutation not observed in outside lab, verified by Sanger sequencing.

Individual mutations that were identified by the Color test, number of patients with each specific mutation, and whether the result matched previous testing by a commercial laboratory. Columns where the match was listed as “Partial” indicate the Color test identified the original mutation observed by a commercial laboratory and an additional mutation that was not observed. In each case, mutations were not observed by a commercial laboratory due to stated limitation in that laboratory’s test.
